# Effects of Exercise Interventions on Peripheral Brain-Derived Neurotrophic Factor: An Umbrella Meta-Analysis Across Clinical and Healthy Populations

**DOI:** 10.3390/jcm15176733

**Published:** 2026-08-30

**Authors:** Mesut Süleymanoğulları, Cemre Didem Eyipınar, Raul Ioan Muntean, Halil İbrahim Ceylan

**Affiliations:** 1 Department of Physical Education and Sport, Faculty of Sport Sciences, Ağrı Ibrahim Çeçen University, 04100 Ağrı, Türkiye; msuleymanogullari@agri.edu.tr; 2 Department of Physical Education and Sport, Faculty of Sport Sciences, Gaziantep University, 27310 Gaziantep, Türkiye; cemreeyipinar@gantep.edu.tr; 3 Department of Physical Education and Sport, Institute of Health Sciences, Gaziantep University, 27310 Gaziantep, Türkiye; 4 Department of Physical Education and Sport, Faculty of Law and Social Sciences, “1 Decembrie 1918” University of Alba Iulia, 10009 Alba Iulia, Romania; 5 Department of Physical Education of Sports Teaching, Faculty of Sports Sciences, Atatürk University, 25240 Erzurum, Türkiye

**Keywords:** BDNF, exercise, umbrella review, corrected covered area, AMSTAR 2

## Abstract

**Background and Objectives:** Brain-derived neurotrophic factor (BDNF) supports neuronal function and plasticity, but exercise meta-analyses may reuse primary studies and combine distinct outcomes. We evaluated review-level evidence while preventing direct reuse of the same primary-study evidence within pooled syntheses in this umbrella review and meta-analysis. **Methods:** Thirty-nine systematic reviews with quantitative syntheses of peripheral BDNF were assessed. Review–primary-study links were normalized, methodological quality was appraised with AMSTAR 2, and estimates were classified by exercise timing and comparison design. Only reviews meeting the zero-overlap criterion were pooled using restricted maximum likelihood random-effects models with Hartung–Knapp inference. Prediction intervals were calculated; network and dose–response findings were summarized separately. **Results:** The corpus included 507 review–primary-study occurrences representing 289 unique normalized primary-study identities (corrected covered area [CCA], 1.99%). AMSTAR 2 overall confidence was low in eight reviews and critically low in 31. Seven reviews meeting the zero-overlap criterion contributed to the chronic-controlled synthesis, yielding a pooled SMD of 0.85 (95% CI 0.07–1.64; I^2^ = 93.9%; 95% prediction interval −1.20 to 2.91). Acute-controlled effects were inconclusive (four reviews; SMD 0.49, 95% CI −0.07 to 1.05; I^2^ = 53.8%), as were chronic pre–post effects (three reviews; SMD 0.32, 95% CI −0.04 to 0.69; I^2^≈0%). Quality-restricted meta-analysis was not estimable, and small-study-effect tests were not performed because no synthesis set contained at least 10 independent reviews. **Conclusions:** Exercise may increase peripheral BDNF in some chronic-controlled settings, but effects vary substantially. Circulating BDNF does not directly establish central BDNF engagement or enhanced neuroplasticity, and current review-level evidence does not establish a universal response, clinical benefit, superior modality, or optimal dose.

## 1. Introduction

Brain-derived neurotrophic factor (BDNF) contributes to neuronal survival, synaptic remodeling, and activity-dependent plasticity, primarily through tropomyosin receptor kinase B signaling [1,2]. Exercise is biologically plausible as a regulator of BDNF. Human arteriovenous measurements support the release of BDNF from the brain during exercise [3], while experimental evidence implicates lactate–SIRT1 and PGC-1α/FNDC5-related signaling in hippocampal BDNF expression [4].

Nevertheless, circulating BDNF is a peripheral, methodologically sensitive biomarker rather than a direct proxy for central BDNF–TrkB signaling. Although human arteriovenous observations are compatible with cerebral BDNF release during exercise [3], a serum or plasma concentration integrates contributions from blood cells and peripheral tissues and cannot localize its source or establish brain target engagement. Serum BDNF is strongly influenced by platelet storage and release during clotting, whereas plasma BDNF represents a lower-concentration circulating pool with potentially different physiological meaning [5]. Specimen type, blood-processing conditions, sampling time, storage, and assay procedures should therefore be considered when interpreting circulating BDNF findings [5,6].

Consequently, changes in circulating BDNF should not be interpreted as direct evidence of BDNF activity, enhanced central neuroplasticity, cognitive improvement, mood benefit, or neurological recovery.

The evidence base has expanded from broad meta-analyses of acute and chronic exercise [5,7] to population-specific, modality-specific, network, and dose–response syntheses. This breadth creates two important inferential problems. First, reviews address different estimands: an immediate response to a single exercise bout differs biologically from resting BDNF after repeated training, while a controlled post-intervention contrast differs methodologically from an uncontrolled pre–post change. Combining these effects may obscure meaningful biological and methodological differences.

Second, multiple reviews may include the same primary studies. Pooling review estimates that reuse the same primary studies as if they were independent can give reused studies excessive statistical weight and produce spuriously narrow confidence intervals. Guidance for overviews of reviews therefore recommends explicit assessment and management of primary-study overlap [8,9]. Moreover, a low corpus-wide corrected covered area may conceal substantial overlap within review pairs or analytical strata. Dependence should therefore be evaluated among the reviews entering each quantitative synthesis rather than through the overall overlap percentage alone.

The present umbrella review prioritized estimand compatibility and avoidance of direct primary-study reuse over maximizing the number of pooled estimates. We aimed to (1) catalog systematic reviews reporting quantitative exercise–BDNF syntheses; (2) map and quantify primary-study overlap; (3) appraise review methodology using AMSTAR 2; (4) synthesize compatible, pairwise zero-overlap estimates separately for chronic-controlled, acute-controlled, and chronic pre–post questions; and (5) summarize network and dose–response evidence without treating correlated outputs from individual evidence structures as independent observations.

## 2. Materials and Methods

### 2.1. Protocol and Registration

This overview of systematic reviews with meta-analysis examined the effects of exercise on peripheral blood brain-derived neurotrophic factor (BDNF). The study was conducted and reported in accordance with the PRIOR statement, methodological guidance for overviews of reviews, and relevant PRISMA 2020 reporting items [8,9,10].

The protocol was prospectively registered in the International Prospective Register of Systematic Reviews (PROSPERO) on 7 May 2026 (registration ID: CRD420261388638), and the final search was executed on 1 August 2026. No amendments have been made to the information provided at the protocol registration. Before conducting the quantitative syntheses, the analysis plan was refined to distinguish acute responses from chronic adaptations, controlled between-group contrasts from uncontrolled within-group pre–post changes, and conventional pairwise estimates from network and dose–response outputs. Biological specimens were classified as serum, plasma, whole blood, or mixed/not separately reported.

To address non-independence arising from repeated inclusion of the same primary studies across reviews, a zero-primary-study-overlap rule was applied to each second-order pooled analysis. Accordingly, no two reviews contributing to the same pooled estimate were permitted to share a normalized primary-study identity. These amendments did not affect eligibility for the descriptive review corpus; rather, they governed the classification of estimands and the selection of review-level estimates eligible for quantitative pooling. All amendments and their methodological rationale are reported transparently in Supplementary Table S1.

### 2.2. Eligibility Criteria

Reports were eligible if they (1) were peer-reviewed systematic reviews with an explicit literature search; (2) quantitatively synthesized data from at least two human primary studies; (3) evaluated structured exercise or exercise training; and (4) reported at least one separately identifiable quantitative synthesis of peripheral blood BDNF.

No restrictions were imposed on exercise modality, participant age, health status, or clinical condition. BDNF concentrations measured in serum, plasma, or whole blood were eligible. Biological matrices were classified at the review-estimate level as serum, plasma, whole blood, or mixed/not separately reported and were not assumed to be interchangeable. When a review supplied matrix-specific effects, these were retained as distinct estimates. However, most eligible review-level effects either combined serum and plasma primary studies or did not report matrix-specific pooled effects; therefore, separate serum-only and plasma-only umbrella meta-analyses were not estimable. Mixed/not separately reported estimates could enter an analysis set only when otherwise compatible and were flagged as a source of biological and analytical heterogeneity.

Eligible comparison structures included controlled between-group contrasts and uncontrolled within-group pre–post changes. Controlled contrasts could compare exercise with non-exercise, usual care, inactive, or other eligible comparison conditions, provided that the relevant exercise contrast was separately identifiable. Reviews including both exercise and non-exercise interventions were eligible only when exercise-related findings could be distinguished from other intervention components.

Narrative or scoping reviews without quantitative synthesis, animal or in vitro studies, reviews reporting only central nervous system BDNF, and reviews of non-exercise interventions without a separately identifiable exercise component were excluded. Acute responses and chronic adaptations were treated as distinct exposure-timing estimands. Controlled between-group contrasts and uncontrolled within-group pre–post changes were treated as distinct comparison estimands and were not combined within a common pooled estimate. Conventional pairwise, network, and dose–response syntheses were classified separately.

Eligibility for each second-order pooled analysis was assessed separately. Review-level estimates were pooled only when they addressed sufficiently compatible estimands with respect to exposure timing, comparison structure, biological specimen, effect definition, and synthesis type, and when no pair of contributing reviews shared any normalized primary-study identity. Reviews not included in a particular pooled analysis because of estimand incompatibility or primary-study overlap remained eligible for the descriptive corpus, methodological appraisal, and overlap analyses.

### 2.3. Search Strategy and Information Sources

A comprehensive systematic search was conducted from database inception on 1 August 2026 across eight electronic databases: PubMed/MEDLINE, Web of Science Core Collection, Scopus, Embase, the Cochrane Library, PsycINFO, SPORTDiscus, and CINAHL.

The search strategy combined controlled vocabulary, where available, with free-text terms representing three concepts: (1) peripheral BDNF, including “brain-derived neurotrophic factor” and “BDNF”; (2) exercise, including “exercise” and “physical exercise”; and (3) evidence type, including “meta-analysis” and “systematic review.” Terms within each concept were combined using the Boolean operator OR, and the three concepts were combined using AND. Search syntax was adapted to the controlled vocabulary, field codes, and indexing conventions of each database.

### 2.4. Study Selection

**Figure 1:**
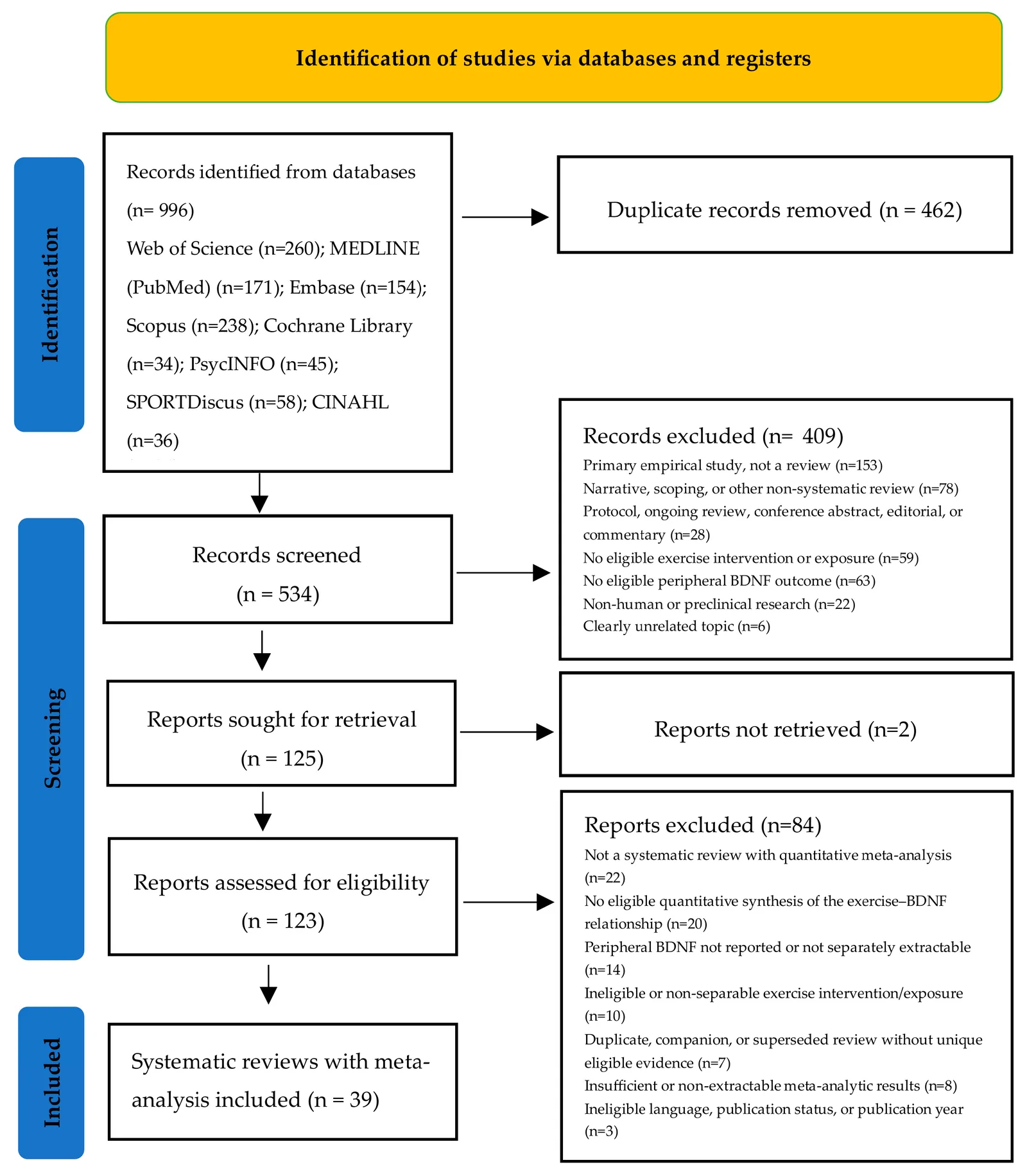
Preferred reporting items for systematic reviews and meta-analyses (PRISMA) flow chart of the study selection process [10].

Records identified through the search were imported into Zotero (version 9.0.2) for deduplication and screening. Two reviewers (MS, HIC) independently screened titles and abstracts against the predefined eligibility criteria and subsequently assessed the full texts of potentially eligible reports. Disagreements at either stage were resolved through discussion, with a third reviewer (CDE) consulted when consensus could not be reached. Reasons for full-text exclusion are reported in the PRISMA flow diagram (Figure 1).

### 2.5. Data Extraction

Data were extracted using a structured form developed from the registered protocol and piloted on three reviews. Extraction was performed independently by two reviewers (MS, HİC), with discrepancies resolved through discussion and, when necessary, adjudication by a third reviewer (CDE). Extracted information included bibliographic characteristics; population and clinical condition; exercise modality; acute or chronic exposure; study design and comparator; BDNF specimen and, when reported, blood-collection timing relative to exercise, clotting or anticoagulant conditions, processing and centrifugation, storage, and assay platform; numbers of primary studies and participants; effect-size metric and pooled estimate; confidence or credible interval; heterogeneity statistics; statistical model; and methodological quality. A source location was retained for each quantitative estimate, and extracted numerical values were checked against the corresponding source reports. Incompletely reported specimen and laboratory characteristics were used for classification and interpretation but could not support formal moderation without enough comparable independent estimates.

### 2.6. Methodological Quality Assessment and Umbrella-Level Certainty

Methodological quality was appraised using AMSTAR 2 [11] by two reviewers (MS, HİC) working independently; disagreements were resolved through discussion, with a third reviewer (CDE) consulted when necessary. All 16 domains, including the seven critical domains, were assessed individually. No numerical summary score was calculated. Overall confidence was classified as high, moderate, low, or critically low according to AMSTAR 2 guidance.

Certainty was assessed for each prespecified synthesis set using an adapted umbrella-level certainty framework informed by GRADE principles [12] and methodological considerations for certainty assessment in umbrella reviews [13]. Because certainty-assessment approaches for umbrella reviews are not standardized [14], the framework was designed specifically for the umbrella-review context rather than to provide conventional primary-study-level GRADE certainty. The assessment considered review-level methodological limitations, inconsistency, indirectness and design limitations, imprecision, small-study effects when estimable, and prediction intervals. AMSTAR 2 ratings were used only to characterize methodological limitations of the contributing reviews/meta-analyses and were not interpreted as measures or surrogates of risk of bias in the underlying primary studies. Certainty was downgraded according to the cumulative severity of limitations across domains; very serious limitations in key domains or multiple serious limitations generally resulted in low or very low umbrella-level certainty. Ratings were assigned independently by two reviewers, with disagreements resolved by discussion or third-reviewer adjudication. The resulting ratings represent the certainty of the second-order synthesis within each prespecified synthesis set, rather than conventional GRADE certainty of the underlying primary-study evidence.

### 2.7. Primary-Study Overlap Strategy

Primary-study overlap was assessed to identify repeated use of the same underlying primary evidence across the included systematic reviews. Primary-study identities were normalized using bibliographic information, including study identifiers, author names, publication year, and study title, with manual verification where necessary. The corrected covered area (CCA) was calculated to quantify overall overlap, and pairwise overlap patterns were examined to identify locally concentrated reuse across groups of reviews. A sensitivity analysis using an author–year matching rule was also performed to assess the robustness of the overall CCA estimate.

Bibliographic overlap was not interpreted as evidence of either conceptual or statistical independence: reviews with little or no shared primary evidence may address closely related research questions, whereas reviews sharing primary studies cannot be assumed to provide independent evidence. Accordingly, CCA and pairwise overlap analyses were used to characterize patterns of evidence reuse rather than to establish statistical independence, methodological quality, certainty, or effectiveness. For quantitative synthesis, primary-study overlap was evaluated at the review-pair level after normalization. The zero-primary-study-overlap criterion was used as a conservative operational approach to prevent direct reuse of the same primary-study evidence within a pooled synthesis and was not interpreted as proof of complete statistical independence.

### 2.8. Analysis Sets and Synthesis Strategy

Following estimand classification and overlap assessment, five analysis sets were defined before model fitting (Supplementary Table S1). S1 comprised chronic-controlled effects, S2 acute-controlled effects, and S3 chronic uncontrolled pre–post effects. These sets contained sufficiently compatible estimands and satisfied the prespecified zero-primary-study-overlap criterion and were therefore eligible for random-effects umbrella pooling. S4 comprised correlated exercise-modality estimates from one Bayesian network meta-analysis in older adults, and S5 comprised model-based dose–response estimates from one Bayesian network meta-analysis in people after a stroke. Because estimates originating from the same network model are correlated, S4 and S5 were summarized descriptively and were not re-pooled as independent umbrella-level observations.

### 2.9. Statistical Analysis

For S1–S3, effect directions were harmonized so that positive values indicated higher peripheral BDNF with exercise. Standard errors were obtained from source reports or derived from 95% confidence intervals when necessary. Only sufficiently compatible standardized mean differences were pooled. Review-level estimates were synthesized using random-effects models with restricted maximum likelihood estimation of between-review variance (τ^2^) and Hartung–Knapp inference [15]. We reported the pooled standardized mean difference with its 95% confidence interval and *p*-value, Cochran’s Q, I^2^, τ^2^, and a 95% prediction interval. In this umbrella review, heterogeneity statistics describe variation among review-level estimates rather than among the primary studies within each review.

Leave-one-review-out analyses were conducted for sets containing at least four reviews. A quality-restricted sensitivity analysis excluding critically low AMSTAR 2 reviews required at least two eligible reviews to remain. Small-study-effect tests were only planned for sets containing at least 10 independent reviews; none met this threshold. Moderator analyses and meta-regression were not conducted because the number of compatible independent reviews was insufficient in each set. S4 and S5 were summarized using their source-reported effect measures and 95% credible intervals. Analyses were conducted in R software (version 4.4.1; R Foundation for Statistical Computing, Vienna, Austria) using the metafor package version 4.8-0 [16]. All statistical tests were two-sided, with *p* < 0.05 considered statistically significant.

## 3. Results

### 3.1. Literature Search and Study Selection

As shown in Figure 1, the database searches identified 996 records. After removal of 462 duplicate records, 534 unique records underwent title and abstract screening, of which 409 were excluded. The full texts of 125 potentially eligible reports were sought for retrieval; two could not be retrieved, leaving 123 reports for full-text eligibility assessment. Of these, 84 were excluded for prespecified reasons, and 39 systematic reviews reporting quantitative syntheses of peripheral blood BDNF outcomes were included in the umbrella meta-analysis.

### 3.2. Characteristics of Included Meta-Analyses

Table 1 summarizes the 39 systematic reviews with quantitative syntheses of peripheral BDNF. The reviews covered healthy adults, adolescents, older adults, and clinical populations, including individuals with stroke, Parkinson’s disease, multiple sclerosis, mild cognitive impairment, dementia, major depressive disorder, obesity, and type 2 diabetes. Exercise interventions included aerobic, resistance, combined or multicomponent, high-intensity interval, sprint interval, traditional Chinese, taekwondo, and other skill-based modalities.

The reviews differed substantially in their target estimands and study designs. Some evaluated acute BDNF responses to a single exercise bout, whereas others examined chronic adaptations following exercise training. Controlled between-group effects, uncontrolled pre–post changes, and mixed contrasts were all represented. Two recent reviews used Bayesian network-based approaches: one evaluated exercise-modality networks in older adults [36], whereas the other used network dose–response models in people after stroke [53]. The number of unique primary studies contributing to an eligible BDNF synthesis ranged from 2 to 47 per review. BDNF was measured in serum, plasma, whole blood, or combinations thereof, although specimen type was not consistently reported. Most review-level estimates combined serum and plasma data or did not provide matrix-specific pooled effects; therefore, separate serum-only and plasma-only second-order meta-analyses were not estimable. Reporting of blood-collection timing, clotting or anticoagulant conditions, centrifugation, storage, and assay platform was also too inconsistent for moderator analysis. Effect measures included standardized mean differences, Hedges’ g, Cohen’s d, raw mean differences, and network-model estimates with credible intervals. This clinical, methodological, and statistical diversity supported separating evidence by acute or chronic exposure and controlled or pre–post design rather than calculating one pooled effect across all reviews.

### 3.3. Corpus and Overlap

The 39 included reviews were published between 2015 and 2026 and covered healthy adolescents and adults, older adults, and populations with depression, obesity, type 2 diabetes, mild cognitive impairment, multiple sclerosis, Parkinson disease, stroke, and other neurological or cardiometabolic conditions. Exercise modalities included aerobic/endurance, resistance, interval, combined or multicomponent, mind–body, aquatic, coordination, and martial-arts-based programs.

**Figure 2:**
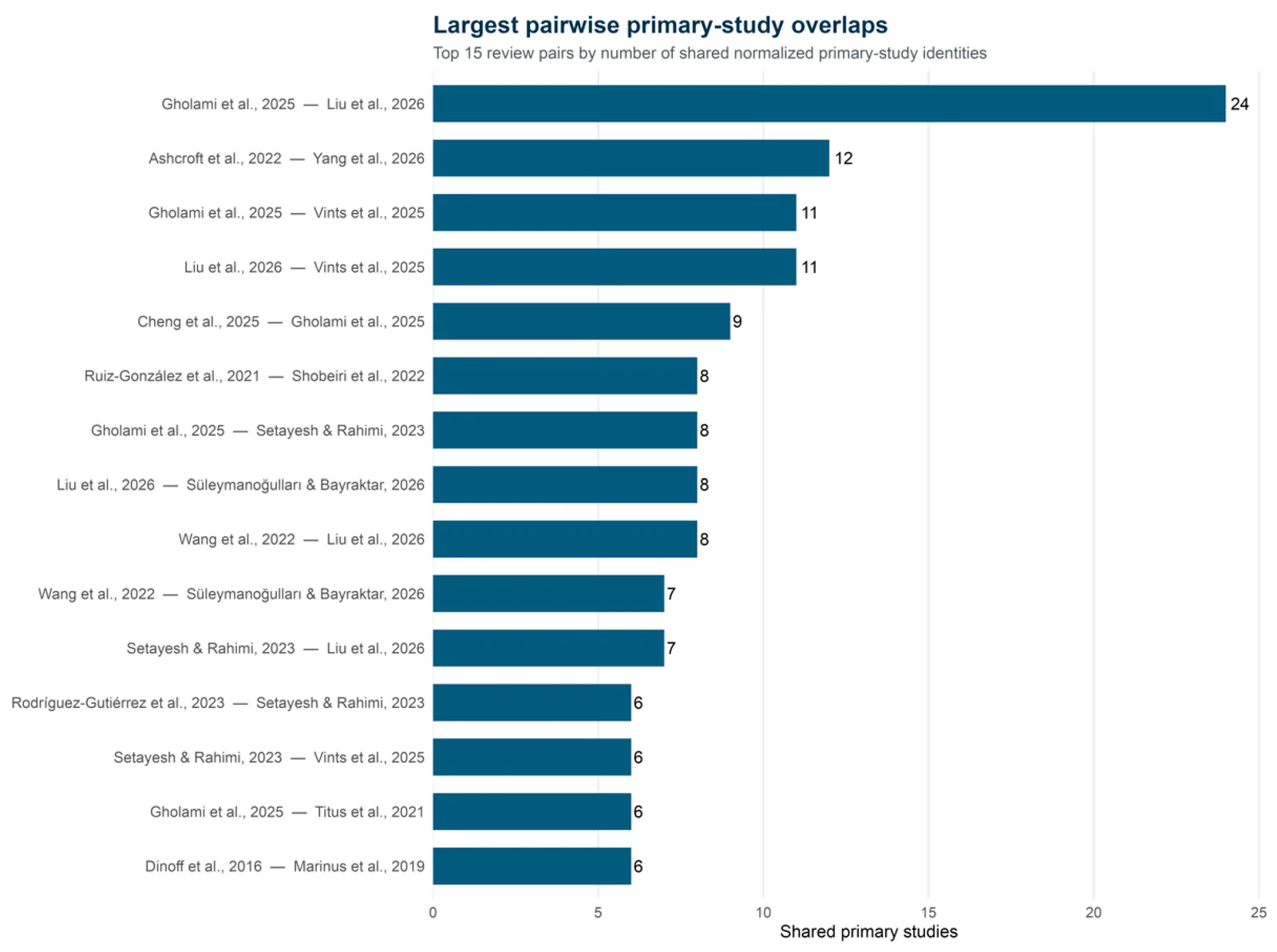
Fifteen review pairs with the largest numbers of shared normalized primary-study identities [5,19,22,30,36,39,42,44,45,46,48,50,51,52,53].

Across the 39 reviews, 507 review–primary-study occurrences represented 289 conservatively normalized primary-study identities. The overall CCA was 1.99%, classified as slight overlap. A sensitivity analysis using the author–year matching approach yielded a CCA of 2.16%. Overlap was nevertheless locally concentrated: 184 review pairs shared at least one primary-study identity, and the largest pair shared 24. The overall CCA therefore did not fully capture locally concentrated overlap evident in the pairwise matrix. The largest pairwise overlap occurred between Gholami et al. [30] and Liu et al. [36], which shared 24 normalized primary-study identities; the next largest pair shared 12. The 15 review pairs with the greatest shared-study counts are shown in Figure 2.

### 3.4. Methodological Quality

**Figure 3:**
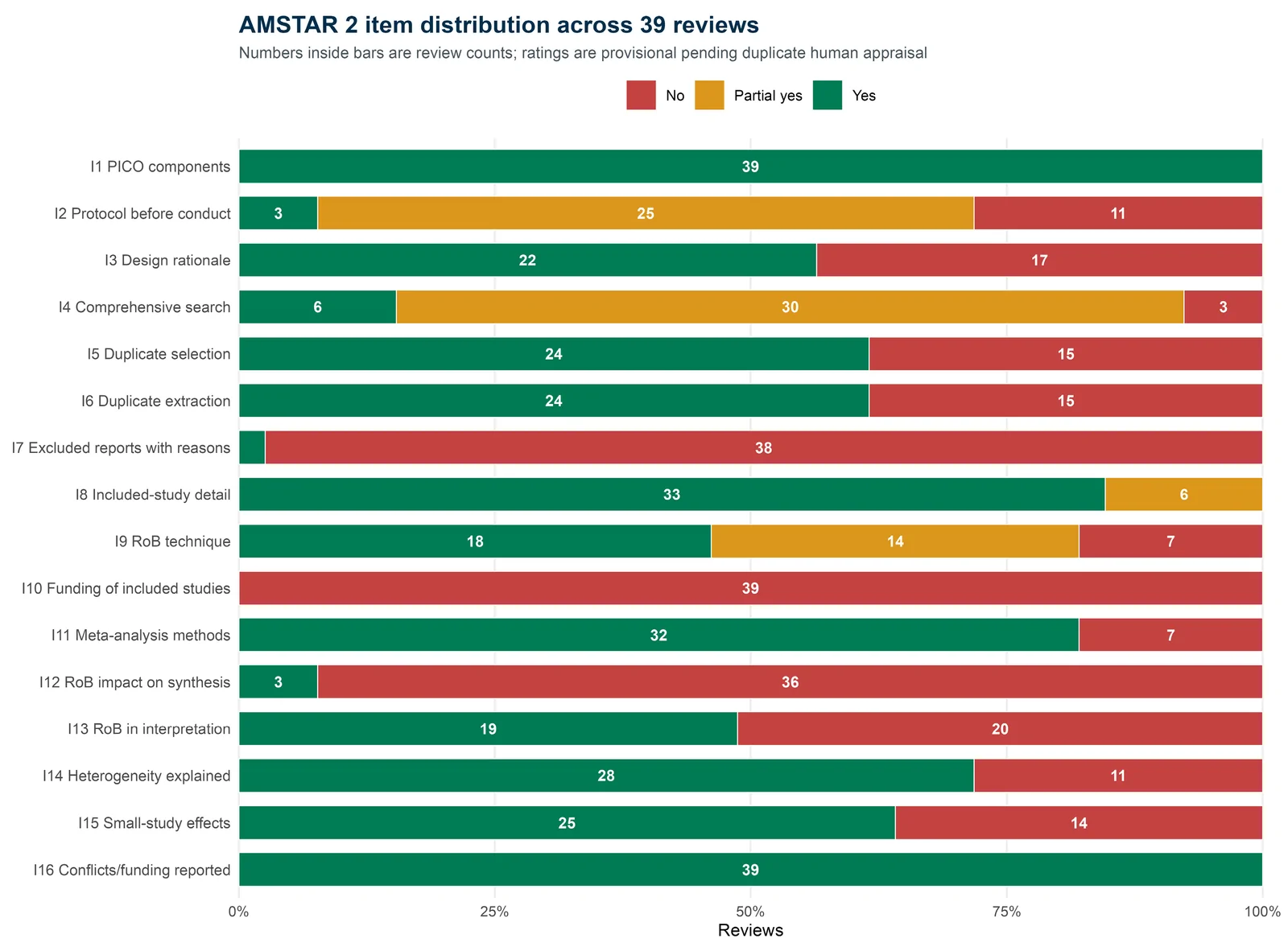
Distribution of AMSTAR 2 item-level judgements across the 39 included reviews. Green, amber, and red segments represent “Yes,” “Partial yes,” and “No” judgements, respectively. Items I2, I4, I7, I9, I11, I13, and I15 constitute the seven AMSTAR 2 critical domains.

AMSTAR 2 assessments indicated substantial methodological limitations across the included evidence base. Overall confidence was rated as low for 8 of the 39 reviews (20.5%) and critically low for 31 (79.5%); no review achieved moderate or high confidence. A study-level list of excluded full-text reports with individual reasons was absent from 38 reviews, and none reported the funding sources of the primary studies they included. Twenty reviews did not adequately incorporate primary-study risk-of-bias assessments into the interpretation of their findings, and 14 did not adequately investigate or discuss small-study effects. Additional limitations included incomplete prospective specification of review methods and weaknesses in duplicate study selection or data extraction. The distribution of all 16 AMSTAR 2 item judgements is presented in Figure 3, the seven critical domains are summarized in Table 2, and the adapted umbrella-level certainty assessments are presented in Table 3.

### 3.5. Primary-Study Overlap

Across the 39 included reviews, 507 review–primary-study occurrences represented 289 unique normalized primary-study identities. The corrected covered area (CCA) was 1.99%, indicating slight overall overlap. An author–year sensitivity analysis yielded a CCA of 2.16%, with the same overall classification of slight overlap. Pairwise overlap analysis identified 184 review pairs with at least one shared primary-study identity, with a maximum of 24 shared studies between any pair. The low overall CCA indicated limited aggregate overlap, although some review pairs showed more pronounced overlap.

### 3.6. Small-Study Effects and Certainty

No pooled synthesis contained at least 10 independent review estimates. Funnel plots and statistical assessments commonly used to investigate small-study effects and potential publication bias, including Egger regression, Begg’s test, trim-and-fill analysis, and fail-safe N, were therefore not performed. Consequently, small-study effects were considered not assessable rather than present or absent.

Using the prespecified adapted synthesis-level certainty framework, certainty was rated as very low for the chronic-controlled (S1), acute-controlled (S2), chronic pre–post (S3), and stroke dose–response (S5) evidence, and as low for the modality-network evidence in older adults (S4). The main reasons for reduced certainty were the low or critically low methodological confidence of the contributing reviews, small numbers of independent estimates, design-related or transdiagnostic indirectness, imprecision, substantial heterogeneity or wide prediction intervals, and the inability to conduct quality-restricted syntheses.

### 3.7. Chronic-Controlled Evidence (S1)

Seven mutually non-overlapping review estimates represented major depressive disorder [23], stroke [34], mild cognitive impairment [37], obesity [21], adolescents [24], type 2 diabetes [32], and healthy adults [48]. The pooled SMD was 0.85 (95% CI 0.07 to 1.64; *p* = 0.0367). Heterogeneity was considerable (τ^2^ = 0.606; I^2^ = 93.9%; Q = 50.38, df = 6, *p* < 0.000001), and the prediction interval ranged from −1.20 to 2.91. At the review level, this interval indicates that a future comparable true effect could plausibly range from a decrease to a large increase. The positive pooled mean therefore does not demonstrate a consistent response across populations or settings, nor does it establish consistency at the individual level.

**Figure 4:**
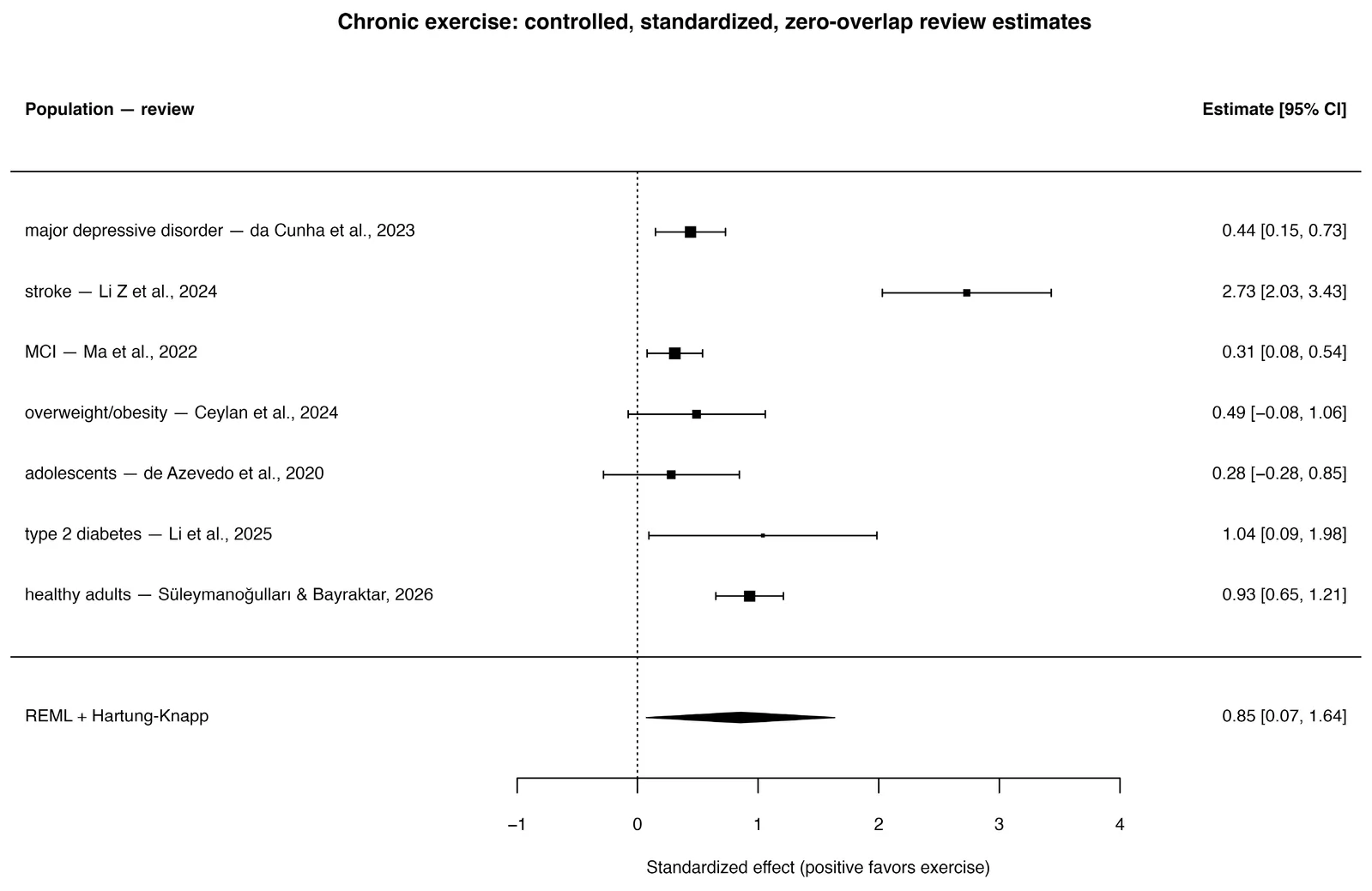
Forest plot for chronic-controlled standardized effects. Notes. Square size reflects inverse-variance weight within the random-effects model; the diamond represents the pooled estimate [21,23,24,32,34,37,48].

Leave-one-review-out point estimates ranged from 0.54 to 0.96, but four of seven confidence intervals crossed zero. Excluding the largest stroke review estimate [34] reduced the pooled SMD to 0.54 (95% CI 0.23 to 0.85). Excluding the healthy-adult review [48] yielded an SMD of 0.85 (95% CI −0.13 to 1.83). Only one S1 review was rated low rather than critically low; therefore, the planned quality-restricted meta-analysis was not estimable. Detailed model estimates are presented in Table 4 and Figure 4.

### 3.8. Acute-Controlled Evidence (S2)

**Figure 5:**
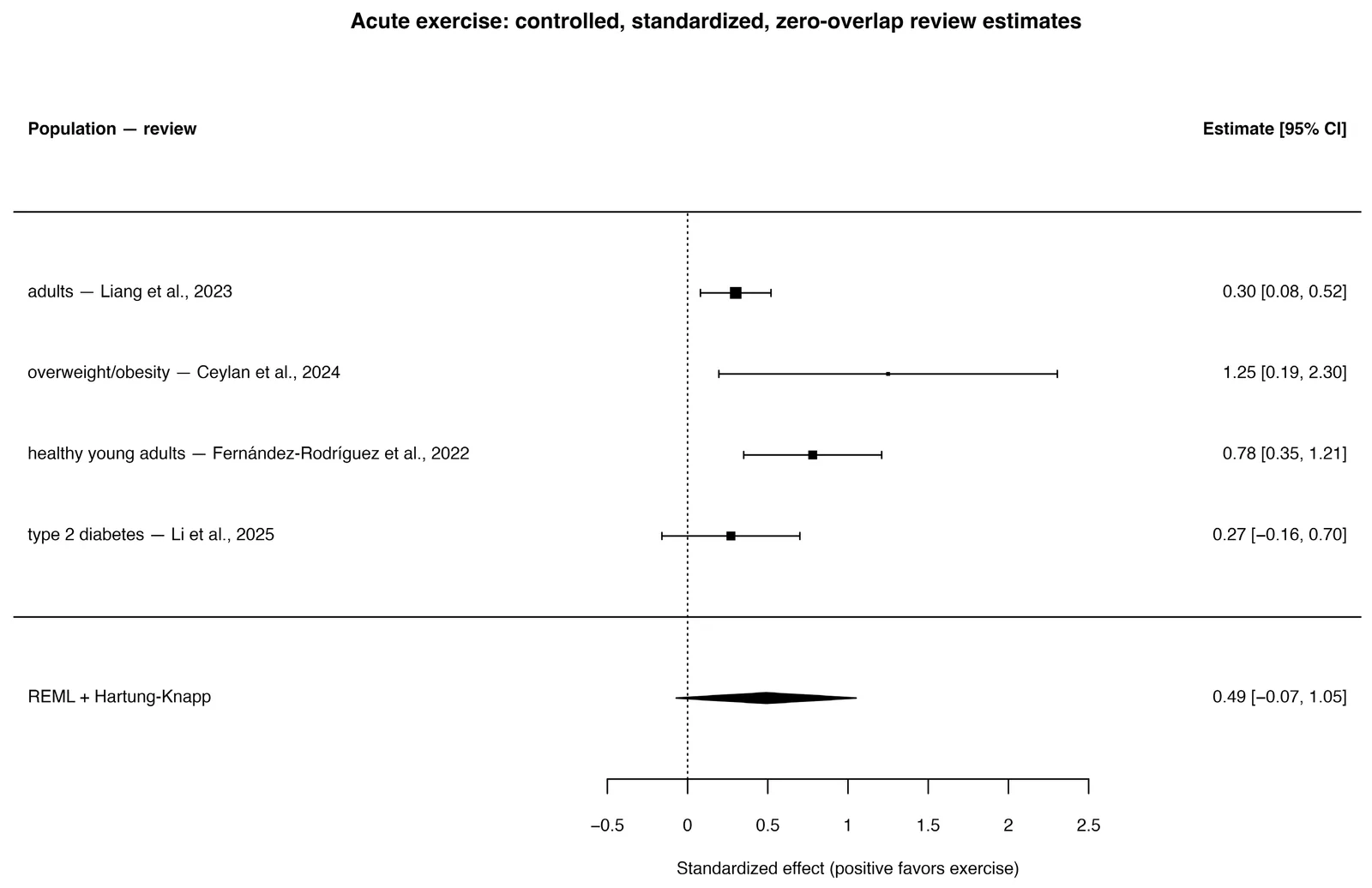
Forest plot for acute-controlled standardized effects. Notes. Square size reflects inverse-variance weight within the random-effects model; the diamond represents the pooled estimate [21,26,32,35].

Four zero-overlap review estimates represented single-bout endurance exercise [35], exercise in people with obesity [21], high-intensity exercise in healthy young adults [26], and exercise in people with type 2 diabetes [32]. The pooled SMD was 0.49 (95% CI −0.07 to 1.05; *p* = 0.0691), with τ^2^ = 0.052, I^2^ = 53.8%, and a prediction interval from −0.43 to 1.41 (Table 4, Figure 5). Leave-one-review-out point estimates ranged from 0.33 to 0.64, and all corresponding confidence intervals crossed zero. Only one review was not rated critically low, precluding the quality-restricted sensitivity analysis.

### 3.9. Chronic Pre–Post Evidence (S3)

**Figure 6:**
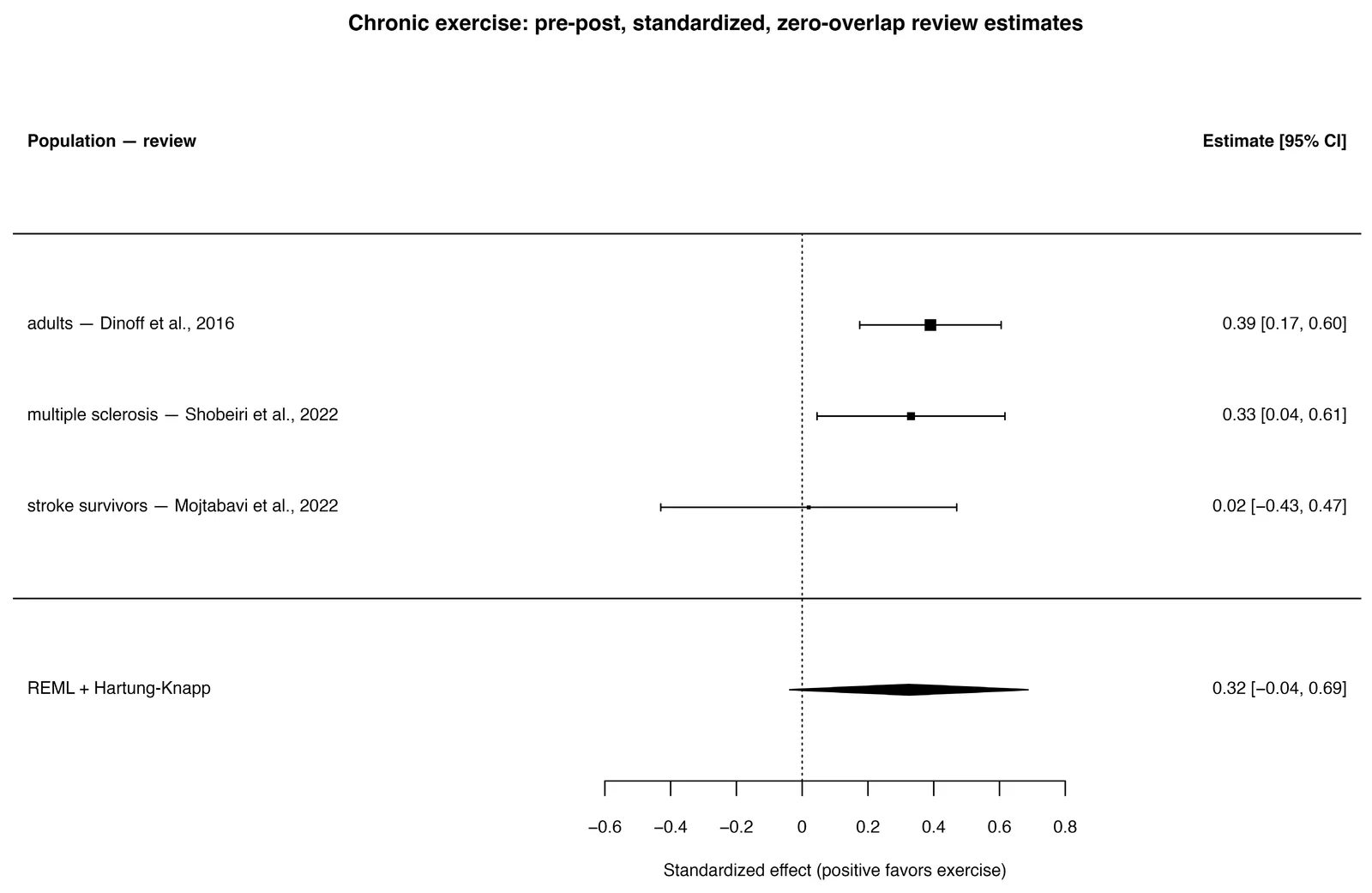
Forest plot for chronic pre–post standardized effects. Notes. Square size reflects inverse-variance weight within the random-effects model; the diamond represents the pooled estimate [5,40,46].

Three zero-overlap reviews synthesized uncontrolled or predominantly pre–post evidence in adults [6], people with multiple sclerosis [46], and people after stroke [40]. The pooled SMD was 0.32 (95% CI −0.04 to 0.69; *p* = 0.0614), with negligible estimated between-review heterogeneity and a prediction interval of −0.04 to 0.69 (Table 4, Figure 6). All three reviews were rated critically low. Because these designs cannot separate an intervention effect from time, regression to the mean, co-interventions, or measurement drift, S3 was interpreted as supportive descriptive evidence rather than causal evidence.

### 3.10. Network and Dose–Response Evidence

A 2026 Bayesian network meta-analysis in older adults [36] reported eight correlated exercise-modality estimates. Aquatic exercise had the largest point estimate (Hedges’ g 1.00, 95% credible interval 0.44 to 1.56), followed by mind–body exercise (0.61, 0.34 to 0.89) and combined aerobic and resistance exercise (0.58, 0.29 to 0.87). Resistance, mixed, and aerobic exercise estimates were smaller but compatible with positive effects, whereas the credible intervals for high-intensity interval training and coordination exercise included zero. The review emphasized the sparsity of some network nodes and the need for cautious interpretation of modality rankings. These correlated estimates were not re-pooled at the umbrella level.

A separate 2026 Bayesian network dose–response meta-analysis in people after stroke [53] reported an acute prediction at 250 MET-min/week of 0.418 (95% credible interval −0.744 to 1.683), a long-term prediction at 1300 MET-min/week of 0.294 (0.007 to 0.615), and a long-term multicomponent prediction at 1700 MET-min/week of 0.984 (0.031 to 1.930). These model-based estimates were interpreted cautiously, were not treated as independent pairwise effects, and were not used to derive clinical exercise prescriptions. The source-model estimates and their credible intervals are summarized in Table 5.

## 4. Discussion

### 4.1. Principal Findings

This overlap-controlled umbrella meta-analysis produced a more restrained conclusion than a conventional second-order pooling of all available review estimates. The chronic-controlled set showed a nominally positive transdiagnostic average, but heterogeneity was extreme, and the prediction interval encompassed both decreases and increases in peripheral BDNF. Acute-controlled and chronic pre–post syntheses were not statistically conclusive. These estimands represent different physiological questions: acute exercise may produce a transient response that changes rapidly with sampling time, whereas chronic training asks whether resting or post-intervention BDNF adapts after repeated exposure. Their explicit separation is therefore a methodological and interpretive strength, and neither should be used interchangeably as evidence of exercise-induced neuroplasticity.

Moreover, “chronic exercise” is not a single biological exposure. Modality, intensity, session duration, program duration, baseline training status, age, sex, adiposity, and metabolic health may modify the observed response. The small number of independent review estimates prevented reliable meta-regression, so these factors remain hypotheses rather than demonstrated umbrella-level moderators.

The low overall CCA did not eliminate the need for overlap management. The corpus-wide CCA was 1.99%, conventionally indicating slight overlap. However, 184 review pairs shared at least one primary study, and the most overlapping pair shared 24 studies, demonstrating that a low corpus-wide CCA can conceal concentrated pairwise overlap. Requiring zero pairwise overlap within each pooled set was conservative and reduced the number of analyzable reviews, but it prevented direct reuse of the same primary-study evidence within a pooled estimate.

### 4.2. Biological and Measurement Interpretation

The positive average is biologically plausible in light of evidence that exercise can influence BDNF-related biological processes [3,4]. Yet the outcome synthesized in these reviews was a peripheral blood concentration rather than central BDNF–TrkB signaling. Serum measurements are strongly shaped by platelet release during clotting, whereas plasma measurements reflect a different peripheral pool [5]. In the present corpus, reporting of clotting or anticoagulant conditions, processing and centrifugation, storage, assay platform, calibration, and sampling relative to exercise was too inconsistent for formal moderator analysis. Because most source reviews combined matrices or reported insufficient matrix-specific results, this heterogeneity could not be separated at the umbrella level.

Importantly, a change in peripheral BDNF is not itself evidence of improved cognition, mood, disability, or neurological recovery. The large stroke estimate contributing to S1 came from a broader rehabilitation review with only three BDNF studies [34]. Its removal materially reduced the pooled average. Consequently, the present results should be interpreted as biomarker evidence and should not be translated directly into clinical treatment effects.

### 4.3. Modality and Dose

The newer network evidence suggests that several modalities may increase BDNF in older adults, with aquatic and mind–body exercise producing relatively large point estimates [36]. However, network rankings are especially vulnerable to sparse nodes, unequal evidence geometry, and violations of transitivity assumptions. Similarly, the stroke dose–response review suggested positive long-term estimates at selected modeled doses [53], but its acute credible interval was wide, and the long-term estimates were predictions from a model incorporating heterogeneous designs. These findings are hypothesis-generating and do not justify identifying a best modality or prescribing a specific dose threshold.

### 4.4. Methodological Quality of the Evidence Base

The absence of moderate- or high-certainty synthesis sets is an important characteristic of the evidence base. AMSTAR 2 does not recommend calculating a summed score; rather, its overall confidence ratings are determined by weaknesses in predefined critical domains [11]. The near-universal absence of a study-level list of excluded reports and the complete absence of information on the funding sources of included primary studies substantially limited the transparency and verifiability of the reviews. Many reviews also did not explain how primary-study risk-of-bias judgements influenced their synthesis or conclusions. Importantly, AMSTAR 2 evaluates the methodological conduct and reporting of systematic reviews; it does not directly assess the risk of bias of the underlying primary studies or the certainty of a specific intervention effect. Accordingly, AMSTAR 2 findings were not used as surrogates for primary-study risk of bias. They were interpreted as review-level methodological limitations within the adapted umbrella-level certainty framework and as indicators of limitations in the transparency and methodological conduct of the evidence base.

### 4.5. Strengths and Limitations

Strengths include comprehensive full-text assessment of potentially eligible reports, source-verified extraction of 108 quantitative estimates, independent numerical verification, normalization of 289 primary-study identities, a traceable citation matrix, explicit separation of biologically and methodologically distinct estimands, conservative zero-overlap pooling, Hartung–Knapp inference, prediction intervals, and transparent handling of network and dose–response outputs. The review authored by members of the review team that contributed to S1 was also examined in a leave-one-review-out sensitivity analysis.

Several limitations should be considered. First, the unit of quantitative synthesis remained the review-level estimate. Even after eliminating primary-study overlap within each synthesis set, the contributing reviews differed in eligible designs, effect-size construction, blood-sampling time, specimen, processing and assay procedures, and primary-study risk of bias. Separate serum and plasma umbrella syntheses and formal analyses of sampling, processing, or assay methods were not feasible because most source reviews mixed matrices or incompletely reported these characteristics. Second, the chronic-controlled synthesis combined clinically diverse populations and therefore represents a broad transdiagnostic average with substantial indirectness. Third, each pooled synthesis contained few independent reviews, limiting inferential precision, the stability of between-review heterogeneity estimates and prediction intervals, and the feasibility of small-study-effect tests, moderator analyses, and quality-restricted syntheses. Available review-level characteristics were insufficient to evaluate modality, intensity, session or program duration, training status, age, sex, adiposity, and metabolic health as moderators; important sources of variability therefore remain unidentified. Finally, overlap estimates depend on primary-study identity normalization. Although the author–year sensitivity analysis produced a similar CCA estimate, residual under-merging or over-merging of study identities cannot be excluded.

### 4.6. Implications for Research and Practice

Future primary trials should prespecify whether the estimand is an immediate acute response, recovery kinetics, or a chronic resting adaptation; collect blood at standardized and, for acute studies, repeated post-exercise time points; and report specimen type, clotting time or anticoagulant, processing delay, centrifugation protocol, storage conditions, assay manufacturer and platform, lot information, and coefficients of variation. Exercise modality, intensity, session duration, program duration, adherence, and baseline training status should also be reported consistently. Trials should provide change and post-intervention data sufficiently for standardized re-analysis and pair peripheral biomarkers with clinical or cognitive outcomes without treating BDNF as a surrogate for these outcomes. Reviews should publish complete study-level exclusion lists, report funding of included primary studies, integrate risk of bias into interpretation, and make extraction and analytical code sufficiently transparent and reproducible. A de-duplicated first-order meta-analysis could then use more consistent estimands and more defensible moderator analyses.

Future trials should prospectively examine potential sources of inter-individual variability. Exercise modality and intensity, program duration, BDNF Val66Met polymorphism, biological sex, menstrual or menopausal status, age, training history, baseline BDNF concentration, adiposity, metabolic health, and circadian timing warrant evaluation as potential effect modifiers [54]. Preliminary human findings suggest that genotype- and sex-related differences in exercise-responsive BDNF may exist, while circulating BDNF concentrations also vary according to hormonal status [55,56]. However, these observations remain insufficient to support genotype- or sex-specific exercise recommendations. Future studies should therefore prespecify these variables, ensure adequate representation across relevant subgroups, and distinguish confirmatory interaction analyses from exploratory subgroup comparisons.

From a broader clinical perspective, exercise remains relevant for its established general health and rehabilitation benefits; however, the present BDNF synthesis should not be used alone to select a modality, intensity, or weekly MET target.

Mechanistic studies would benefit from measuring BDNF alongside lactate, irisin, IGF-1, VEGF, inflammatory markers, and standardized indices of exercise dose and fitness. Human multimarker studies are feasible [56,57], and experimental work supports lactate-dependent BDNF signaling [4]; however, these findings do not establish that any circulating biomarker mediates the neurological or cognitive effects of exercise. Peripheral BDNF measurements should consequently be linked prospectively with cognition, mood, physical function, neurological recovery, and structural or functional neuroimaging outcomes. A randomized trial in older adults, for example, related exercise-associated changes in serum BDNF to hippocampal volume and memory outcomes, illustrating the value of integrated biomarker–neuroimaging designs without validating peripheral BDNF as a surrogate clinical endpoint [58]. Future research should move beyond asking whether exercise raises circulating BDNF on average and test who responds, under which exercise conditions, and, using prespecified mediation analyses, whether the BDNF response mediates meaningful neuropsychological or metabolic outcomes.

## 5. Conclusions

Across 39 eligible reviews, the chronic-controlled, zero-overlap synthesis showed a positive pooled estimate for the effect of exercise on peripheral BDNF. Still, the estimate was highly heterogeneous, subject to substantial methodological limitations, and associated with a prediction interval spanning both decreases and increases. Acute-controlled and chronic pre–post findings were inconclusive and address distinct physiological questions. Network and dose–response findings were informative but derived from single-correlated evidence structures and remained hypothesis-generating. Overall certainty was low or very low. The most defensible conclusion is conditional: exercise may modify peripheral BDNF in some settings, but current review-level evidence cannot establish a universal biomarker response, central BDNF engagement or enhanced neuroplasticity, mediation of clinical benefit, a best modality, or an optimal dose.

## Figures and Tables

**Table 1:** Characteristics of the 39 included quantitative systematic reviews.

Review	Population	Exercise Scope	BDNF Estimand/Design	Unique Primary BDNF Studies, k	Specimen	Effect Measure
Akalp et al., 2024 [17]	Older adults with mild cognitive impairment	Mixed exercise	Chronic; controlled	3	Serum/plasma	SMD
Andreu-Caravaca et al., 2025 [18]	Adults with multiple sclerosis	Predominantly aerobic exercise	Acute and short-term; pre–post	8	Serum/plasma	SMD
Ashcroft et al., 2022 [19]	Stroke survivors	Aerobic and non-aerobic exercise	Acute and chronic; mixed designs	16	Serum/plasma	MD
Behrad et al., 2024 [20]	Healthy older adults	Aerobic, resistance, and multicomponent exercise	Chronic; pre–post	11	Serum/plasma	Standardized mean effect
Ceylan et al., 2024 [21]	People with overweight or obesity	Mixed exercise	Acute and chronic; controlled	14	Serum/plasma	Hedges’ g
Cheng et al., 2025 [22]	Older adults	Walking, running, and cycling	Chronic; controlled	15	Serum/plasma	SMD/Hedges’ g
da Cunha et al., 2023 [23]	Adults with major depressive disorder	Predominantly aerobic exercise	Chronic; controlled	4	Circulating blood; type varied or NR	SMD
de Azevedo et al., 2020 [24]	Adolescents	Aerobic exercise	Chronic; controlled RCTs	2	Serum	SMD
Dinoff et al., 2016 [5]	Healthy and clinical adult populations	Aerobic and resistance exercise	Chronic; pre–post	28	Serum/plasma	SMD
Farrukh et al., 2023 [25]	Women aged ≥60 years	Aerobic, power, aquatic, taekwondo, and multimodal exercise	Chronic; controlled	8	Serum/plasma	Cohen’s d/MD
Fernández-Rodríguez et al., 2022 [26]	Healthy young adults	High-intensity interval and continuous exercise	Acute; controlled/pre–post	10	Serum/plasma	Standardized p-ES/MD
Fleitas et al., 2022 [27]	Sedentary older adults	Aerobic and resistance exercise	Chronic; controlled/pre–post	10	Serum/plasma	SMD
Gan et al., 2025 [28]	Middle-aged and older adults	Traditional Chinese exercise	Chronic; controlled RCTs	9	Serum/plasma	Hedges’ g
García-Suárez et al., 2021 [29]	Healthy young adults	HIIT and sprint interval training	Acute and chronic; mixed contrasts	15	Serum/plasma	SMD
Gholami et al., 2025 [30]	Older adults	Aerobic, resistance, and combined exercise	Chronic; controlled	35	Serum/plasma	SMD
Kurebayashi and Otaki, 2018 [31]	Adults with major depressive disorder	Predominantly aerobic/mixed exercise	Chronic; controlled	5	Serum/plasma	SMD
Li et al., 2025 [32]	Middle-aged and older adults with type 2 diabetes	Aerobic, resistance, and combined exercise	Acute and chronic; controlled	9	Serum/plasma	SMD
Li X et al., 2024 [33]	Older adults	Taekwondo	Chronic; controlled	4	Serum	SMD
Li Z et al., 2024 [34]	Stroke survivors	Moderate/vigorous aerobic exercise	Chronic; controlled	3	Peripheral blood; type NR	SMD
Liang et al., 2023 [35]	Adults	Endurance exercise	Acute; randomized controlled	8	Serum/plasma	SMD
Liu et al., 2026 [36]	Older adults	Eight exercise-modality classes	Chronic; Bayesian network meta-analysis	47	Serum/plasma	Hedges’ g
Ma et al., 2022 [37]	Adults with mild cognitive impairment	Aerobic, resistance, and combined exercise	Chronic; controlled	7	Serum/plasma	SMD
Mackay et al., 2017 [38]	People with neurological disorders	Aerobic exercise	Chronic; controlled	5	Serum	SMD
Marinus et al., 2019 [39]	Older adults	Aerobic, resistance, and combined exercise	Acute and chronic; controlled/pre–post	17	Serum/plasma	SMD
Mojtabavi et al., 2022 [40]	Stroke survivors	Exercise/rehabilitation	Acute and delayed; pre–post	7	Serum/plasma	SMD
Qipo et al., 2026 [41]	Older adults	Resistance training	Chronic; controlled	2	Serum/plasma	Hedges-adjusted SMD
Rodríguez-Gutiérrez et al., 2023 [42]	Adults aged ≥45 years without cognitive impairment	Resistance exercise	Chronic; controlled RCTs	7	Serum/plasma	Cohen’s d
Rotondo et al., 2023 [43]	People with Parkinson’s disease	Aerobic, multicomponent, and skill-based exercise	Chronic; controlled	5	Serum/plasma	MD
Ruiz-González et al., 2021 [44]	People with neurodegenerative disorders	Mixed exercise	Chronic; controlled RCTs	17	Serum/plasma	SMD
Setayesh and Rahimi, 2023 [45]	Adults aged ≥60 years	Resistance training	Chronic; controlled RCTs	11	Serum/plasma	MD
Shobeiri et al., 2022 [46]	People with multiple sclerosis	Mixed exercise	Chronic; pre–post	12	Serum/plasma	Hedges’ g
Stigger et al., 2019 [47]	Older adults with mild cognitive impairment or dementia	Aerobic and combined exercise	Chronic; controlled	3	Serum/plasma	MD
Süleymanoğulları and Bayraktar, 2026 [48]	Healthy adults	Aerobic, resistance, and combined exercise	Chronic; controlled RCTs	20	Serum	SMD
Süleymanoğulları et al., 2026 [49]	Neurological and non-neurological clinical populations	Aerobic, resistance, and combined exercise	Chronic; controlled RCTs	19	Serum/plasma	SMD
Szuhany et al., 2015 [7]	Healthy and clinical adult populations	Mixed exercise	Acute and chronic; mixed contrasts	29	Serum/plasma	Hedges’ g
Titus et al., 2021 [50]	Older adults	Predominantly aerobic/mixed exercise	Chronic; controlled	8	Serum/plasma	SMD
Vints et al., 2025 [51]	Older adults	Mixed exercise	Chronic; three-level controlled meta-analysis	30	Serum/plasma	SMD
Wang et al., 2022 [52]	Healthy participants	Mixed exercise	Acute and chronic; randomized	21	Serum/plasma	SMD
Yang et al., 2026 [53]	Stroke survivors	Acute and long-term exercise modalities	Acute and chronic; Bayesian network/dose–response	23	Serum/plasma	Hedges’ g

Abbreviations: BDNF, brain-derived neurotrophic factor; HIIT, high-intensity interval training; k, number of unique primary studies contributing to at least one eligible peripheral BDNF synthesis within each review (comparison arms and repeated subgroup estimates were not counted as separate studies); MD, mean difference; NR, not reported; RCT, randomized controlled trial; SMD, standardized mean difference; p-ES, pre–post effect size. In the specimen column, “serum/plasma” denotes a review including both matrices and does not provide separable matrix-specific pooled estimates; it does not imply biological equivalence.

**Table 2:** Distribution of AMSTAR 2 critical-domain judgements.

Item	Critical Domain	Yes, *n*	Partial Yes, *n*	No, *n*
I2	Protocol established before conduct of the review	3	25	11
I4	Comprehensive literature search strategy	6	30	3
I7	List of excluded full-text reports with reasons	1	0	38
I9	Appropriate technique for assessing risk of bias	18	14	7
I11	Appropriate methods for statistical combination	32	0	7
I13	Consideration of risk of bias when interpreting results	19	0	20
I15	Adequate investigation and discussion of small-study effects	25	0	14

Note: Counts are based on 39 reviews. Overall confidence was rated as low for eight reviews (20.5%) and critically low for 31 (79.5%); no review achieved moderate or high confidence. AMSTAR 2 judgements were not converted into a numerical summary score.

**Table 3:** Adapted umbrella-level certainty assessment for the prespecified synthesis sets.

Synthesis Set	Evidence Summary	Review-Level Limitations	Inconsistency	Indirectness and Design-Related Limitations	Imprecision	Small-Study Effects	Umbrella-Level Certainty
S1: Chronic-controlled	Seven reviews; SMD 0.85 (95% CI 0.07 to 1.64); 95% PI −1.20 to 2.91	Very serious; six reviews were critically low, and one was low by AMSTAR 2	Serious; I^2^ = 93.9% and effects varied substantially	Serious; transdiagnostic synthesis across clinically different populations	Serious; wide CI and prediction interval spanning decreases and increases	Not assessable; k < 10	Very low
S2: Acute-controlled	Four reviews; SMD 0.49 (95% CI −0.07 to 1.05); 95% PI −0.43 to 1.41	Very serious; three reviews were critically low, and one was low by AMSTAR 2	Serious concern; I^2^ = 53.8% with only four reviews	Some concern because populations and acute exercise protocols differed	Serious; confidence and prediction intervals crossed the null	Not assessable; k < 10	Very low
S3: Chronic pre–post	Three reviews; SMD 0.32 (95% CI −0.04 to 0.69); 95% PI −0.04 to 0.69	Very serious; all three reviews were critically low	No important statistical heterogeneity detected, but estimation was limited by k = 3	Very serious; uncontrolled pre–post changes do not identify a causal intervention effect	Serious; confidence interval crossed the null	Not assessable; k < 10	Very low
S4: Modality network	One network review; eight correlated modality estimates in older adults	Serious; evidence originated from one review and several network nodes were sparse	Not assessable at the umbrella level because all estimates came from one network	Limited to older adults; rankings depended on network assumptions and evidence geometry	Serious for several modalities; some credible intervals crossed the null	Not assessable at the umbrella level	Low
S5: Stroke dose–response network	One network review; three selected model-based dose predictions	Serious; evidence originated from one review	Not assessable at the umbrella level	Serious; estimates were model-based predictions across heterogeneous designs and doses	Serious; credible intervals were wide, particularly for the acute estimate	Not assessable at the umbrella level	Very low

Note. Certainty was assessed using an adapted umbrella-level certainty framework informed by GRADE principles and methodological considerations for umbrella reviews [12,13]. Ratings represent the certainty of the second-order synthesis within each prespecified synthesis set and should not be interpreted as conventional GRADE certainty ratings for the underlying primary-study evidence. AMSTAR 2 ratings were only used to characterize methodological limitations at the review level and were not treated as measures or surrogates of primary-study risk of bias. Small-study effects were classified as not assessable because no quantitative synthesis set contained at least 10 independent review estimates.

**Table 4:** Overlap-controlled pooled results.

Set	k	Pooled SMD (95% CI)	*p*	τ^2^	I^2^ (%)	95% Prediction Interval	Certainty
S1 chronic-controlled	7	0.85 (0.07 to 1.64)	0.037	0.606	93.9	−1.20 to 2.91	Very low
S2 acute-controlled	4	0.49 (−0.07 to 1.05)	0.069	0.052	53.8	−0.43 to 1.41	Very low
S3 chronic pre–post	3	0.32 (−0.04 to 0.69)	0.061	<0.001	≈0	−0.04 to 0.69	Very low

Notes. All models used REML with Hartung–Knapp inference. Positive values indicate higher peripheral BDNF with exercise.

**Table 5:** Network and dose–response evidence summarized without umbrella re-pooling.

Set	Source	Target/Node	Effect (95% CrI)	Interpretive Status
S4	Liu et al., 2026 [36]	Eight exercise-modality nodes in older adults	g range 0.11 to 1.00	Correlated network estimates; umbrella-level certainty: low
S5	Yang et al., 2026 [53]	Acute, 250 MET-min/week	0.418 (−0.744 to 1.683)	Model prediction; interval includes zero
S5	Yang et al., 2026 [53]	Long term, 1300 MET-min/week	0.294 (0.007 to 0.615)	Model prediction; hypothesis-generating
S5	Yang et al., 2026 [53]	Long-term multicomponent, 1700 MET-min/week	0.984 (0.031 to 1.930)	Model prediction; hypothesis-generating

Notes. g, Hedges’ g; CrI, credible interval. S4 and S5 estimates originate within single-correlated Bayesian evidence structures and were not treated as independent umbrella-level effects.

## Data Availability

The data presented in this study are available on request from the corresponding author.

## Supplementary Materials

The following supporting information can be downloaded at: https://www.mdpi.com/article/10.3390/jcm15176733/s1, Supplementary Table S1: Analysis sets and synthesis rules.
