# Supplementary material for: Effects of Exercise Interventions on Peripheral Brain-Derived Neurotrophic Factor: An Umbrella Meta-Analysis Across Clinical and Healthy Populations
Source: J Clin Med. 2026 Aug 30;15(17):6733. doi: 10.3390/jcm15176733 (PMC13565808; doi:10.3390/jcm15176733)
Supplement: Supplementary file 1 [file jcm-15-06733-s001.zip › jcm-4527624-supplementary.pdf]

Supplementary Table S1. Analysis sets and synthesis rules

Set	Analysis question	Evidence included	Synthesis method	Interpretive role
S1: Chronic controlled	Chronic exercise versus a control condition	7 reviews	Random-effects REML model with Hartung–Knapp inference	Primary exploratory synthesis; zero pairwise primary-study overlap
S2: Acute controlled	Acute exercise versus a control condition	4 reviews	Random-effects REML model with Hartung–Knapp inference	Secondary exploratory synthesis; zero pairwise primary-study overlap
S3: Chronic pre–post	Change from before to after chronic exercise without an independent controlled contrast	3 reviews	Random-effects REML model with Hartung–Knapp inference	Secondary exploratory synthesis; uncontrolled evidence retained separately
S4: Modality network	Comparative effects of exercise modalities in older adults	1 network meta-analysis; 8 correlated estimates	Structured narrative and tabular summary	Correlated source-model estimates; no umbrella re-pooling
S5: Dose–response network	Model-based exercise dose–response after stroke	1 network meta-analysis; 3 correlated estimates	Structured narrative and tabular summary	Source-model predictions; no umbrella re-pooling

Abbreviations: REML, restricted maximum likelihood.
